# Tensor- and high-resolution fiber tractography for the delineation of the optic radiation and corticospinal tract in the proximity of intracerebral lesions: a reproducibility and repeatability study

**DOI:** 10.1007/s00701-023-05540-7

**Published:** 2023-03-02

**Authors:** Pavlina Lenga, Moritz Scherer, Peter Neher, Jessica Jesser, Irada Pflüger, Klaus Maier-Hein, Andreas W. Unterberg, Daniela Becker

**Affiliations:** 1 Department of Neurosurgery, Heidelberg University Hospital, Im Neuenheimer Feld 400, 69120 Heidelberg, Germany; 2 German Cancer Research Center, Division of Medical Image Computing, Heidelberg, Germany; 3 Department of Neuroradiology, Heidelberg University Hospital, Heidelberg, Germany; 4 Department of Radiation Oncology, Heidelberg University Hospital, Heidelberg, Germany

**Keywords:** Fiber tracking, Diffusion-tensor imaging, Q-ball imaging, High-resolution fiber tractography, Optic radiation, Corticospinal tract

## Abstract

**Purpose:**

Fiber tracking (FT) is used in neurosurgical planning for the resection of lesions in proximity to fiber pathways, as it contributes to a substantial amelioration of postoperative neurological impairments. Currently, diffusion-tensor imaging (DTI)-based FT is the most frequently used technique; however, sophisticated techniques such as Q-ball (QBI) for high-resolution FT (HRFT) have suggested favorable results. Little is known about the reproducibility of both techniques in the clinical setting. Therefore, this study aimed to examine the intra- and interrater agreement for the depiction of white matter pathways such as the corticospinal tract (CST) and the optic radiation (OR).

**Methods:**

Nineteen patients with eloquent lesions in the proximity of the OR or CST were prospectively enrolled. Two different raters independently reconstructed the fiber bundles by applying probabilistic DTI- and QBI-FT. Interrater agreement was evaluated from the comparison between results obtained by the two raters on the same data set acquired in two independent iterations at different timepoints using the Dice Similarity Coefficient (DSC) and the Jaccard Coefficient (JC). Likewise, intrarater agreement was determined for each rater comparing individual results.

**Results:**

DSC values showed substantial intrarater agreement based on DTI-FT (rater 1: mean 0.77 (0.68–0.85); rater 2: mean 0.75 (0.64–0.81); *p* = 0.673); while an excellent agreement was observed after the deployment of QBI-based FT (rater 1: mean 0.86 (0.78–0.98); rater 2: mean 0.80 (0.72–0.91); *p* = 0.693). In contrast, fair agreement was observed between both measures for the repeatability of the OR of each rater based on DTI-FT (rater 1: mean 0.36 (0.26–0.77); rater 2: mean 0.40 (0.27–0.79), *p* = 0.546). A substantial agreement between the measures was noted by applying QBI-FT (rater 1: mean 0.67 (0.44–0.78); rater 2: mean 0.62 (0.32–0.70), 0.665). The interrater agreement was moderate for the reproducibility of the CST and OR for both DSC and JC based on DTI-FT (DSC and JC ≥ 0.40); while a substantial interrater agreement was noted for DSC after applying QBI-based FT for the delineation of both fiber tracts (DSC > 0.6).

**Conclusions:**

Our findings suggest that QBI-based FT might be a more robust tool for the visualization of the OR and CST adjacent to intracerebral lesions compared with the common standard DTI-FT. For neurosurgical planning during the daily workflow, QBI appears to be feasible and less operator-dependent.

## Introduction

Performing surgery on cerebral lesions in proximity to major white matter tracts is challenging and is regularly supported by technical innovations [5, 13]. Diffusion-weighted (DW) magnetic resonance imaging (MRI)-based fiber tractography (FT) has become indispensable because it enables maximum tumor resection while concurrently preserving the neurological function, contributing to longer survival rates as well as better quality of life in patients [20]. Specifically, the most commonly applied diffusion tensor imaging (DTI)-based FT enables identification and delineation of the course of the eloquent fiber pathways in white matter non-invasively [2, 17]. DTI-based FT is already integrated in common navigation systems and provides user-friendly processing applications; however, a major shortcoming of this technique is due to the fact that only one fiber direction can be resolved within each imaging voxel. Recently, the use of representation techniques, based on high angular resolution diffusion (HARDI) signals, has been introduced as a key tool to mitigate the impediments held by DTI-based FT [7, 9]. In particular, HARDI typically measures diffusion signals along 60 or more gradient directions of the sphere in q-space, enabling, for example, the resolution of intravoxel fiber crossing [10]. However, clinical application of these so-called techniques for HRFT is still restricted by long acquisition times or sophisticated postprocessing [1, 16, 19].

Tracking results depend on the selection of regions of interest (ROI) which are applied to estimate white matter pathways [8, 12] and particularly in cases of manually-selected ROIs, interrater bias is expected. Thus, experience and training in the interpretation of a given anatomical structure is paramount when selecting ROIs. However, little is known about the reproducibility and variability of DTI- and compared with HARDI-based FT for detecting white matter pathways in proximity to cerebral lesions in the clinical setting.

Owing to the lack of robust clinical evidence, we designed this study to examine the intra- and interrater variability of white matter pathway reconstruction using the examples of the corticospinal tract (CST) and optic radiation (OR), by evaluating the dice similarity coefficient (DSC) and Jaccard coefficient (JC) as produced by DTI- and QBI-based FT.

## Methods

### Study design, inclusion, and exclusion criteria

Clinical and imaging data were collected prospectively over a 5-year period (04/2017–02/2022). The present study was conducted in accordance with the Declaration of Helsinki and was approved by the local ethics committee (S-146/2017; S147/2017). Each patient provided written informed consent for participation in the study. Patients aged ≥ 18 years with a suspected intercerebral lesion in the proximity of the OR or the CST were enrolled. In particular, the narrow eligibility criteria were a preoperative MRI protocol including DW sequences by default. The deterrent localization of the lesion was determined as < 20 mm distance from the estimated fiber bundles. Patients younger than 18 years, with incomplete MRI imaging data or general exclusion criteria for MRI were excluded from this study.

### Imaging analysis

Within 3 days before surgery, a 3 Tesla preoperative MRI dataset (on Magnetom Prisma [Siemens, Erlangen, Germany]) was obtained. The following sequences were required for this study: T1-weighted three-dimensional (3D) magnetization-prepared rapid gradient-echo (MPRAGE) þ gadolinium: repetition time (TR) 1790 ms, echo time (TE) 3.7 ms, field of view (FoV) 250 mm, slice thickness 1 mm, 160 slices, sagittal, 3:29 min; fluid-attenuated inversion recovery (FLAIR): TR 8500 ms, TE 136 ms, FoV 230 mm, 25 slices, 2:52 min; diffusion weighted imaging (DWI): TR 6600 ms, TE 87 ms, FoV 256 mm, 56 slices, numbers of excitations I, b = 1000 s/mm^2^, 64 noncollinear diffusion-encoding gradients, voxel size of 2 × 2 × 2 mm^3^, 7:50 min. The total acquisition time was approximately 25 min.

The open-source software MITK Diffusion (https://github.com/MIC-DKFZ/MITK-Diffusion) as part of the Medical Imaging Interaction Toolkit was used for FT [15]. The same DW-MRI sequence was used for both tracking approaches (DTI and QBI). After data transfer into MITK Diffusion, T1-MPRAGE and DW images were rigidly registered. Further preprocessing of the DW images included head motion correction and eddy current correction using affine registration to the unweighted volume. This process was followed by the calculation of tensors using the Insight Toolkit for DTI and dODF for QBI. FT was performed directly on MITK Diffusion for both DTI- and QBI-based fiber reconstruction. The same manually segmented regions of interest (ROI) were applied for the OR, one around the lateral geniculate nucleus (LGN) and the second over the visual cortex (Brodmann areas 17–19). For the CST, included ROIs were drawn in the mesencephalic peduncle and in the precentral gyrus. False positive fibers were excluded with excluded ROIs.

For tractography, sophisticated parameters were used considering common recommendations and comparability: for QBI: sharpen ODFs [10] (recommendation for dODFs in MITK Diffusion); GFA-cutoff, 0.15; step size, 0.5 voxels; angular threshold, 20; minimum tract length, 20 mm; for DTI: FA cutoff, 0.15; step size, 0.5 voxels; angular threshold, 20°; minimum tract length, 20 mm [5].

#### Dice similarity coefficient and Jaccard coefficient

The Dice similarity coefficient (DSC) and Jaccard coefficient (JC) were calculated to examine the reproducibility and similarity of the fiber bundles. The JC is defined as the number of voxels where two ROIs overlap, divided by the number of voxels that any of the two ROIs have included. As a result, the JC ranges from 0, which is no overlap at all, to 1, which is complete agreement [22].

The DSC provides information regarding the tract shape similarity between two data sets. The value of DSC ranges from 0 to 1, with 0 indicating no spatial overlap between two sets of spatial segmentation and 1 indicating complete overlap [24]. As previously proposed by Zijdenbos et al., a good overlap occurs when DSC > 0.700 [24]. Specifically, DSC and JC are also special cases of kappa statistics commonly used in reliability analysis [21].

### Raters

Since the purpose of this analysis was to examine intra- and interrater variability, we decided to use independently obtained data from two different raters. Both raters (one young with 2-year experience and one senior resident with over 10-year experience) were neurosurgeons with experience in neuroimaging diagnostics. Each rater was asked to conduct two FT measurements per case at two timepoints 1 week apart.

### Statistics

Based on kappa statistics, DSC and JC were interpreted as follows: ≤ 0.2 = slight agreement, > 0.2–0.4 = fair agreement, > 0.4–0.6 = moderate agreement, > 0.6–0.8 = substantial agreement, > 0.8 = almost perfect agreement, 1.0 = perfect agreement [21]. An independent *t*-test was applied to test potential differences between raters. The interrater agreement was evaluated from the comparison between results obtained by the two raters on the same data set acquired at round 1 and round 2. Potential statistical differences were tested by applying the Kruskal–Wallis test. Likewise, intrarater agreement was assessed for each rater, comparing individual rounds 1 and 2. All values are given with means and range. A *p*-value < 0.05 was set as statistically significant. We conducted all statistical analyses using SPSS software, Version 22.0.0.0 (IBM Corp., Armonk, NY, USA).

## Results

**Fig. 1:**
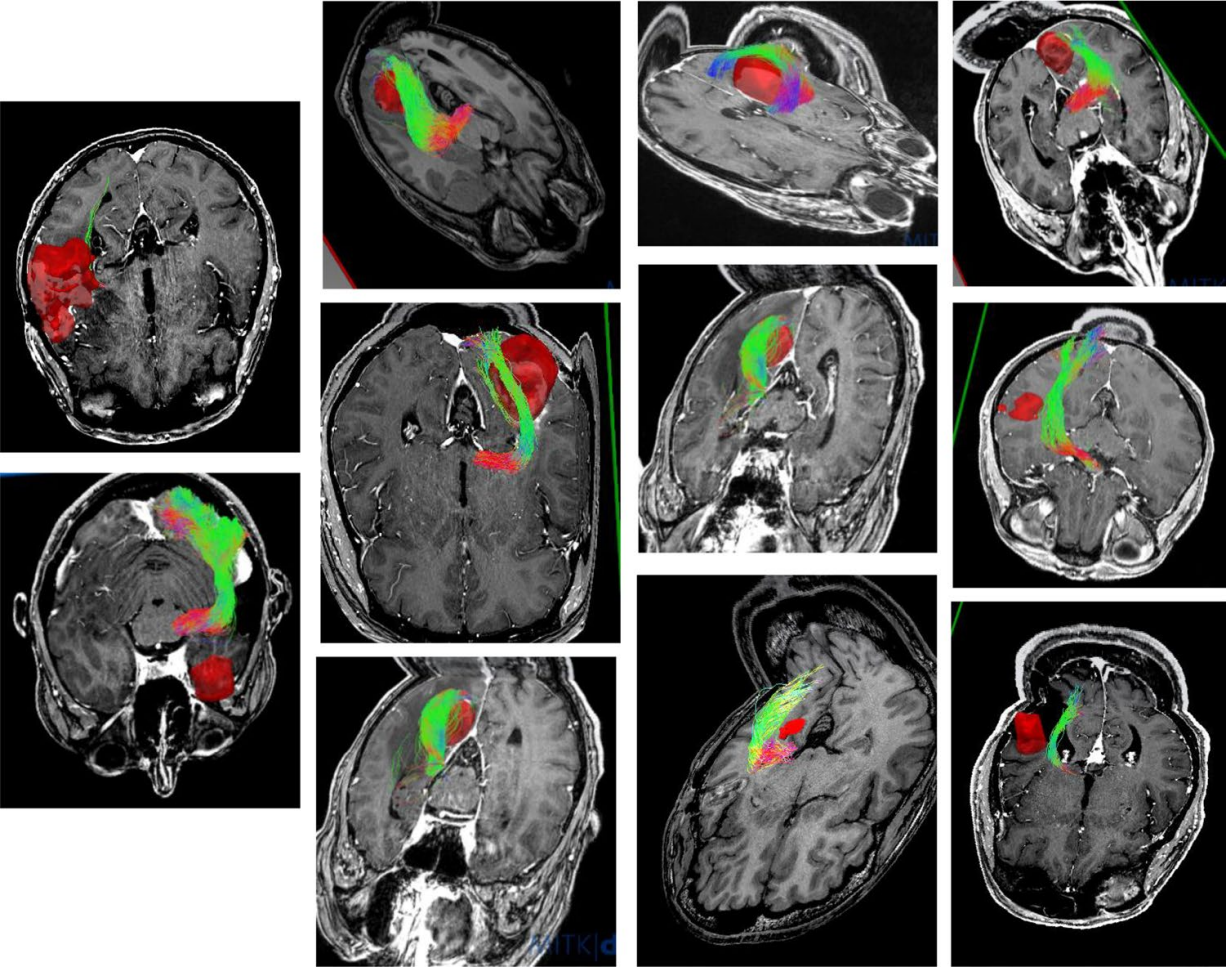
Overview of OR FT results. Tumors displayed in red. MRI T1-weighted, 3D view, cropped. All Rater 1, QBI-FT. (OR, optic radiation; QBI, Q-ball Imaging; FT, fiber tractography)

**Fig. 2:**
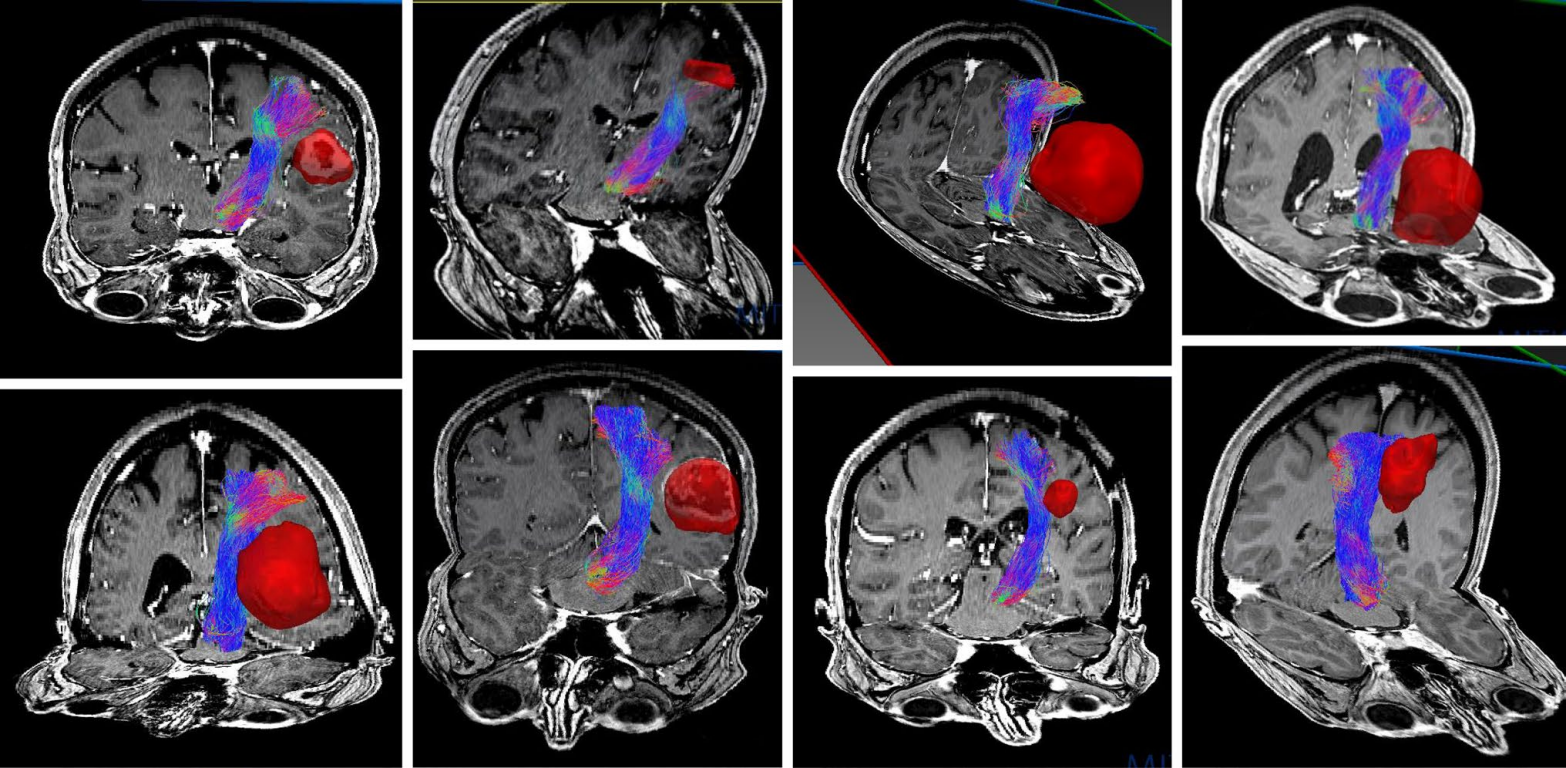
Overview of CST FT results. Tumors displayed in red. MRI T1-weighted, 3D view, cropped. All Rater 1, QBI-FT. (CST, corticospinal tract; QBI, Q-ball Imaging; FT, fiber tractography)

A total of 12 patients with a lesion in the proximity of the OR and 7 patients with a lesion adjacent to the CST were included in the present study. In particular, 7 gliomas in the proximity of the CST and 11 gliomas and 1 cavernoma in the proximity of the OR were analyzed, respectively. An overview of CST and OR in the proximity of the tumor is given by Figs. 1 and 2.

### Technical considerations

The CST processing time between examiners was similar for both applied techniques (DTI processing time, examiner 1: mean 12.5 min SD 1.3 vs examiner 2: 12.7 SD 1.4; *p* = 0.985; QBI processing time, examiner 1: mean 15.5 min SD 1.1 vs examiner 2: 16.7 SD 1.2; *p* = 0.995). No significant differences were obtained for OR (DTI processing time, examiner 1: mean 22.1 min SD 4.3 vs examiner 2: 23.7 SD 5.4; *p* = 0.965; QBI processing time, examiner 1: mean 34.1 min SD 8.1 vs examiner 2: 35.8 SD 9.2; *p* = 0.975).

### Intrarater agreement

The DSC values of repeatability measures showed a substantial agreement between the measurements of the same rater based on DTI-FT findings for the CST [rater 1: mean 0.77 (0.68–0.85); rater 2: mean 0.75 (0.64–0.81)]; while an almost perfect agreement was observed after the deployment of QBI-based FT [rater 1: mean 0.86 (0.78–0.98); rater 2: mean 0.80 (0.72–0.91)]. Of note, after comparing the observations of both raters, no significant differences were observed. A substantial agreement between both measures of each rater was obtained for JC and similarly no significant differences were recorded. A detailed breakdown of the intrarater analysis is provided in Table 1. The same analysis was run for the evaluation of the OR. A fair agreement was observed between both measures of each rater based on DTI-FT [rater 1: mean 0.36 (0.26–0.77); rater 2: mean 0.40 (0.27–0.79), *p* = 0.546], while a substantial agreement between the measures was noted for QBI-FT [rater 1: mean 0.67 (0.44–0.78); rater 2: mean 0.62 (0.32–0.70), *p* = 0.665]. A fair agreement concerning the JC was observed between the measures of each rater. Table 2 demonstrates the findings of the intrarater agreement for the evaluation of the OR.

**Table 1:** Intrarater agreement for evaluation of the corticospinal tract (CST) by applying the Dice similarity coefficient and the Jaccard coefficient

	Mean, range	*p*
Dice similarity coefficient _DTI		0.673
Rater 1	0.77 (0.68–0.85)	
Rater 2	0.75 (0.64–0.81)	
Jaccard coefficient_DTI		0.698
Rater 1	0.64 (0.52–0.73)	
Rater 2	0.66 (0.57–0.73)	
Dice similarity coefficient_QBI		.0693
Rater 1	0.86 (0.78–0.98)	
Rater 2	0.80 (0.72–0.91)	
Jaccard coefficient_QBI		0.150
Rater 1	0.68 (0.43–0.81)	
Rater 2	0.66 (0.32–0.79)

**Table 2:** Intrarater agreement for evaluation of the optic radiation (OR) by applying the Dice similarity coefficient and the Jaccard coefficient

	Mean, range	*p*
Dice similarity coefficient _DTI		0.546
Rater 1	0.36 (0.26–0.77)	
Rater 2	0.40 (0.27–0.79)	
Jaccard coefficient_DTI		0.667
Rater 1	0.25 (0.03–0.31)	
Rater 2	0.27 (0.04–0.38)	
Dice similarity coefficient_QBI		0.598
Rater 1	0.67 (0.44–0.78)	
Rater 2	0.62 (0.32–0.70)	
Jaccard coefficient_QBI		0.665
Rater 1	0.36 (0.10–0.60)	
Rater 2	0.27 (0.09–0.52)	

### Interrater agreement

**Fig. 3:**
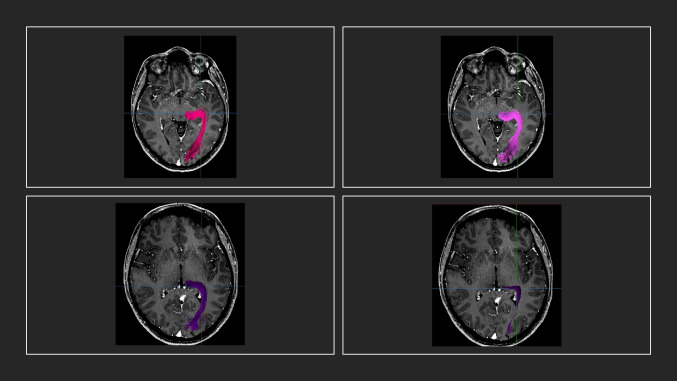
MRI T1-weighted + gadolinium in axial view displaying a temporopolar tumor in the proximity of the OR. Upper line/rater 1: left: OR based on DTI-FT. Right: OR based on QBI-FT. Lower line/rater 2: left: OR based on QBI-FT. Right: OR based on DTI-FT. (MRI, magnetic resonance imaging; OR, optic radiation; QBI, Q-ball Imaging; DTI, Diffusion Tensor Imaging)

**Fig. 4:**
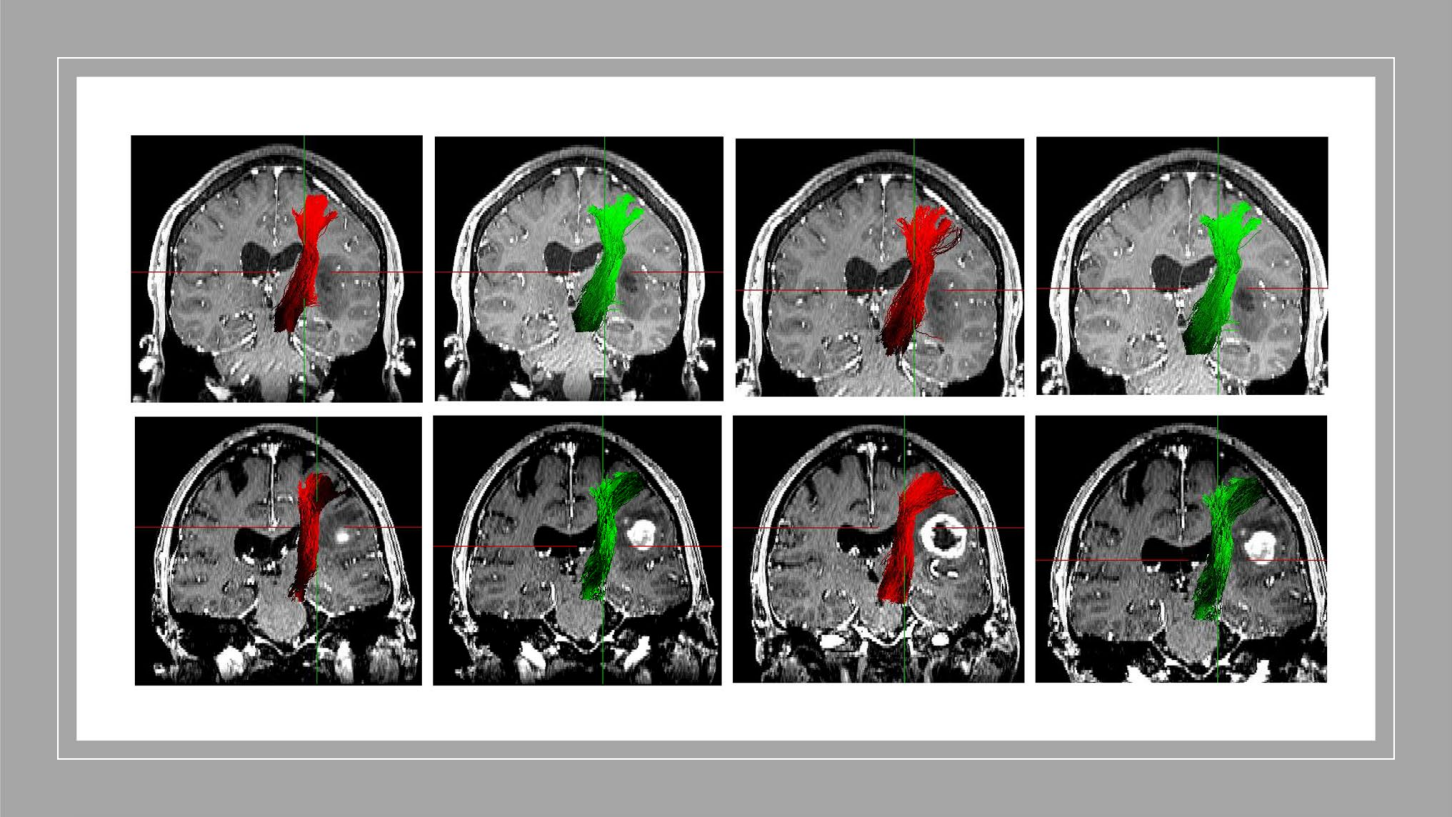
MRI T1-weighted + gadolinium in axial oblique view displaying a non-contrast enhancing temporo-occipital glioma in the proximity of the OR. From left to right: Rater 1, DTI-FT, Rater 1, QBI-FT. Rater 2, DTI-FT, Rater 2, QBI-FT. (MRI, magnetic resonance imaging; OR, optic radiation; QBI, Q-ball Imaging; DTI, Diffusion Tensor Imaging)

**Fig. 5:**
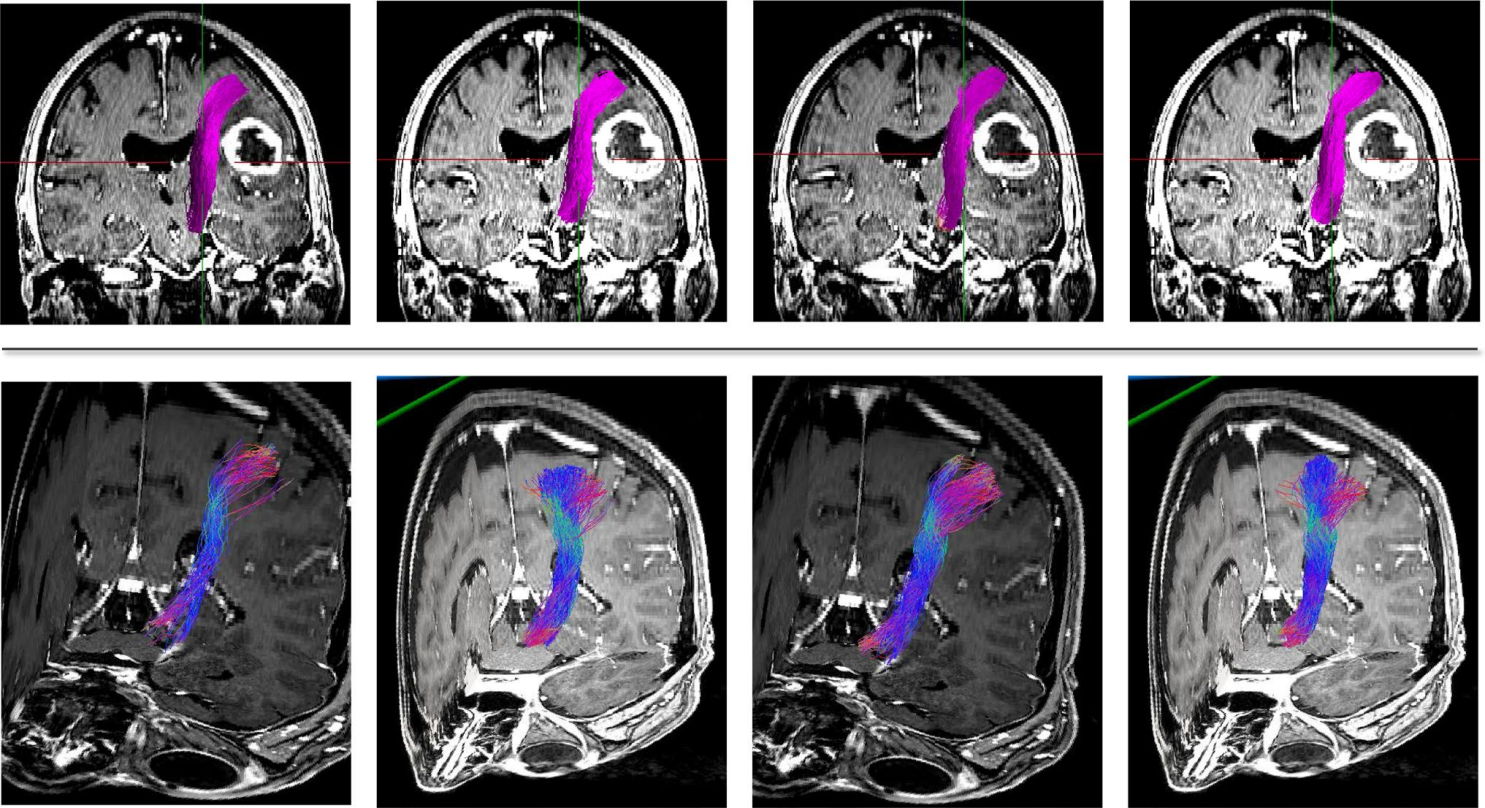
MRI T1-weighted + gadolinium in coronal view depicting the CST as reconstructed by Rater 1. Upper line: patient with non-contrast enhancing fronto-temporo-insular glioma left. From left to right: DTI-FT round 1, DTI-FT round 2. QBI-FT round 1, QBI-FT round 2. Lower line: patient with contrast enhancing glioma left frontal. From left to right: DTI-FT round 1, DTI-FT round 2. QBI-FT round 1, QBI-FT round 2. (MRI, magnetic resonance imaging; CST, Corticospinal Tract; QBI, Q-ball Imaging; DTI, Diffusion Tensor Imaging)

**Fig. 6:**
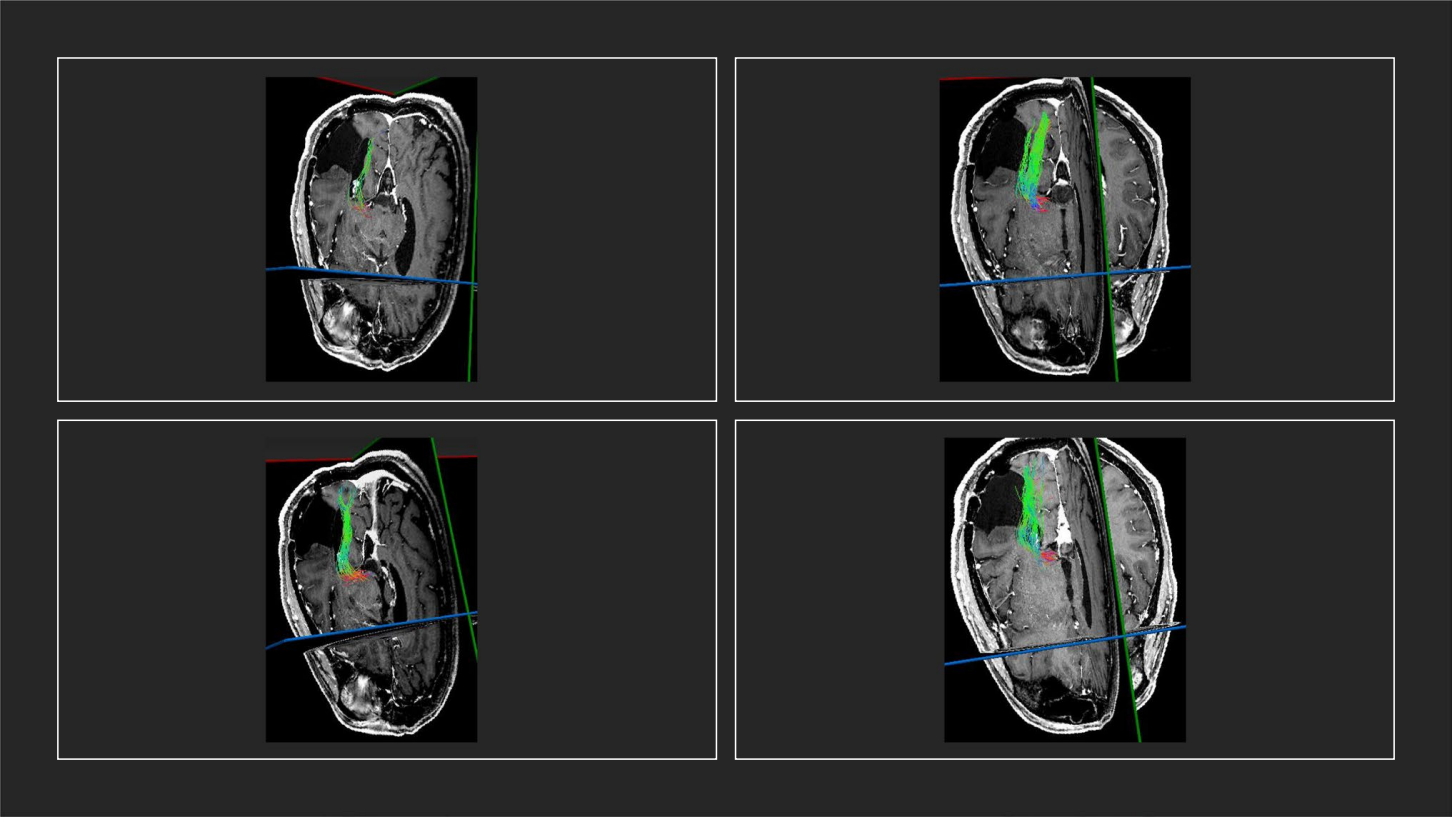
MRI T1-weighted + gadolinium in coronal view depicting the CST as reconstructed by Rater 2. Upper line (coronal view): patient with contrast enhancing frontal glioma left. From left to right: DTI-FT round 1, DTI-FT round 2. QBI-FT round 1, QBI-FT round 2. Lower line (coronal oblique 3D): patient with non-contrast enhancing glioma left frontal. From left to right: DTI-FT round 1, DTI-FT round 2. QBI-FT round 1, QBI-FT round 2. (MRI, magnetic resonance imaging; CST, Corticospinal Tract; QBI, Q-ball Imaging; DTI, Diffusion Tensor Imaging; 3D, three dimensional)

Interrater agreement for the evaluation of the reproducibility of the CST was moderate, as calculated through DSC and JC based on DTI-FT, irrespective of the round of the measures (DSC > 0.40 range 0.23–0.70; JC > 0.40, range 0.13–0.74). In contrast, for QBI-based FT, the interrater agreement was substantial for DSC (DSC > 0.70 range 0.44–0.87) and moderate for JC (JC > 0.40, range 0.30–0.74), as displayed in Table 3, again, irrespective of the round of the measures. Most importantly, no significant differences were observed between the raters and the different timepoints in the respective of the fiber tracking techniques. Figures 3 and 4 display the OR as reconstructed by raters 1 and 2. Similar to the findings of the CST, interrater agreement regarding the DSC based on DTI-FT for the OR was moderate (DSC > 0.40, range 0.20–0.79), irrespective of the round of the measures, while interrater agreement regarding the JC based on DTI-FT was fair (JC > 0.20, range 0.14–0.64). In contrast, the interrater agreement concerning the DSC based on QBI-FT was substantial (DSC > 0.60 range 0.51–0.88), while moderate with regard to JC (JC > 0.40, range 0.31–0.78) (Table 4). Akin to the findings of the CST, no significant differences were observed between both raters with respect to the applied technique. Representative examples after the first and second rounds of each rater for the reconstruction of CST are delineated in Figs. 5 and 6.

**Table 3:** Interrater agreement using the Dice similarity coefficient and the Jaccard coefficient for evaluation of the corticospinal tract (CST)

	Mean, range	*p*
Dice similarity coefficient _DTI		0.410
Rater 1_round 1 vs. Rater 2_round 1	0.51 (0.23–0.70)	
Rater 1_round 1 vs. Rater 2_round 2	0.55 (0.34–0.71)	
Rater 1_round 2 vs. Rater 2_round 1	0.54 (0.33–0.73)	
Rater 1_round 2 vs. Rater 2_round 2	0.58 (0.39–0.70)	
Jaccard coefficient_DTI		0.388
Rater 1_round 1 vs. Rater 2_round 1	0.49 (0.13–0.78)	
Rater 1_round 1 vs. Rater 2_round 2	0.50 (0.21–0.749	
Rater 1_round 2 vs. Rater 2_round 1	0.48 (0.22–0.75)	
Rater 1_round 2 vs. Rater 2_round 2	0.55 (0.17–0.78)	
Dice similarity coefficient_QBI		0.279
Rater 1_round 1 vs. Rater 2_round 1	0.72 (0.44–0.84)	
Rater 1_round 1 vs. Rater 2_round 2	0.73 (0.47–0.87)	
Rater 1_round 2 vs. Rater 2_round 1	0.71 (0.46–0.86)	
Rater 1_round 2 vs. Rater 2_round 2	0.74 (0.55–0.85)	
Jaccard coefficient_QBI		0.118
Rater 1_round 1 vs. Rater 2_round 1	0.55 (0.30–0.75)	
Rater 1_round 1 vs. Rater 2_round 2	0.56 (0.31–0.74)	
Rater 1_round 2 vs. Rater 2_round 1	0.57 (0.32–0.76)	
Rater 1_round 2 vs. Rater 2_round 2	0.57 (0.38–0.74)

**Table 4:** Interrater agreement using the Dice similarity coefficient and the Jaccard coefficient for evaluation of the optic radiation (OR)

	Mean, range	*p*
Dice similarity coefficient _DTI		0.539
Rater 1_round 1 vs. Rater 2_round 1	0.40 (0.20–0.76)	
Rater 1_round 1 vs. Rater 2_round 2	0.42 (0.21–0.77)	
Rater 1_round 2 vs. Rater 2_round 1	0.41 (0.22–0.79)	
Rater 1_round 2 vs. Rater 2_round 2	0.44 (0.32–0.77)	
Jaccard coefficient_DTI		0.487
Rater 1_round 1 vs. Rater 2_round 1	0.27 (0.14–0.61)	
Rater 1_round 1 vs. Rater 2_round 2	0.26 (0.14–0.62)	
Rater 1_round 2 vs. Rater 2_round 1	0.29 (0.16–0.64)	
Rater 1_round 2 vs. Rater 2_round 2	0.32 (0.21–0.62)	
Dice similarity coefficient_QBI		0.732
Rater 1_round 1 vs. Rater 2_round 1	0.64 (0.51–0.87)	
Rater 1_round 1 vs. Rater 2_round 2	0.65 (0.52–0.88)	
Rater 1_round 2 vs. Rater 2_round 1	0.67 (0.53–0.88)	
Rater 1_round 2 vs. Rater 2_round 2	0.64 (0.51–0.87)	
Jaccard coefficient_QBI		0.522
Rater 1_round 1 vs. Rater 2_round 1	0.49 (0.31–0.77)	
Rater 1_round 1 vs. Rater 2_round 2	0.50 (0.41–0.78)	
Rater 1_round 2 vs. Rater 2_round 1	0.52 (0.42–0.77)	
Rater 1_round 2 vs. Rater 2_round 2	0.50 (0.32–0.74)

## Discussion

To the best of our knowledge, we are the first to examine tract-shape similarity and reproducibility by applying the DSC and JC in the OR and CST adjacent to intracerebral lesions using DTI- and QBI-based FT in terms of intra- and interrater agreement in the clinical setting. We found that interrater agreement was substantial when calculating the OR and CST with QBI-based FT, whereas moderate interrater agreement was achieved with DTI-based FT for defining both fiber bundles according to the DSC. Interestingly, DSC and JC showed almost perfect intrarater agreement for QBI-based FT and substantial intrarater agreement for DTI-based FT for the reproducibility of the CST. A substantial agreement between the measures of each rater concerning the DSC was only seen after the deployment of QBI-based FT for the delineation of the OR. In contrast, a moderate agreement was shown for both parameters (DSC and JC) for the display of the OR after the deployment of DTI-based FT.

### Interrater observations

The observed high interrater variability may be explained based on the following factors:

- General variability

The existing literature describes variability in results for both evaluated tracts when reconstructed based on diffusion-weighted images. For the CST, particular variability has been described with regard to DTI’s resolution of the fibers carrying information to the upper extremities and face, as opposed to the leg/foot region. To solve this problem, novel algorithms or techniques were considered, and they provided better results [3]. In particular, the FT of the OR poses a challenge owing to the high curvature of Meyer’s loop (ML) as well as the high variability of individual trajectories. It is noteworthy that this variability may be attributable to ROI selection, given that, as previously described by Benjamin et al., multiple seed regions surrounding the LGN were found to seed streamlines consistent with the known anatomical course of the OR [6]. Opposed to this procedure, we chose a two-ROI approach with manually segmented ROIs delineating the LGN and visual cortex for our specific analysis. Our results suggest that the application of larger included ROIs or more adjacently placed ROIs might reduce the interrater variability.

- Confounding factor: manual ROI placement and changes in neuroanatomical structure

The lower interrater agreement in the present study can be further explained by structural neuroanatomical changes due to the lesion itself or the presence of perilesional edema, affecting manual ROI segmentation, which has been done by examiners with different levels of experience in FT. It is noteworthy, that the manually selected ROIs are the only difference during the fiber tracking procedure comparing raters 1 and 2. Apart therefrom, other default parameters were identical. To further shed light on potential differences among the raters, we performed a univariate analysis aiming to compare the results of both raters with regard to DSC and JC. Interestingly, whereas the raters had different level of experience, we did not find any significant differences between the tractography results of the senior and junior examiner in any comparison (see Tables 3 and 4). However, we strongly believe that the perceptibility of anatomical areas is essential for an FT result, particularly in cases of poor anatomical definition when adjacent to tumor and peritumoral edema. This might be a reason for the observed slight varying of inclusion-, or exclusion ROIs. Different from the OR or CST, this is even more crucial for instance for the reconstruction of the language-associated pathways, for which cortical areas serving as include ROIs are less well defined and which are known to imply higher neuroanatomical plasticity. In these cases, manual ROI segmentation should be supplemented with technical aids to define cortical areas like functional MRI or transcranial magnetic stimulation (TMS), which has been suggested by other study groups [13, 14].

- Robustness of HRFT

While the interrater agreement was moderate for DTI-FT, QBI-FT still provided substantial and thus generally less interrater variability for the given fiber bundles, suggesting more robust results herewith. This might be due to the fact that the tensor model is more sensitive to areas with disturbed diffusion properties such as tumor or edema or deterrent regions with a high amount of intra-voxel fiber crossing (e.g., temporal stem). For example Zhang et al. compared fiber bundles using QBI- and DTI-based FT within the peritumoral edema and advocated that QBI-based FT might be a promising tool, as it enables the visualization of fiber bundles even within the edematous area while DTI-based FT does not [23]. Similarly, Kuhnt et al. showed that the ML could be reconstructed in 50% of the analyzed cases, whereas such results could not be obtained with DTI-based FT in a small cohort of glioma patients with tumors adjacent to the OR.

This is also in line with novel findings comparing DTI- and QBI-FT, suggesting not only more solid and compact fiber bundles [5], but also a better quality of QBI-FT results compared with intraoperative monitoring under awake craniotomy [5]. Particularly for pathways with a neuroanatomical complexity such as the OR, DTI-FT still frequently fails or delivers implausible results. These implausible and false positive fibers might also contribute for the higher interrater variability of DTI-FT.

### Intrarater observation

Substantial to almost perfect intrarater reliability was observed for the visualization of both fiber bundles with both QBI-FT fair to substantial results for DTI-FT (when observing the DSC), which is in line with previously published data exclusively on the reconstruction of the OR [18], showing that intrarater variability is generally lower than interrater variability. However, QBI-FT seems to be even less susceptible compared with DTI-FT. Alltogether, this again emphasizes the impact of ROI placement, assuming that one rater chose similar ROIs in the first and second FT-iteration, which is in line with previously published data [13, 14]. Also, less experienced raters produce comparable results with almost perfect intrarater variability, at least with QBI-FT. Nonetheless, the quality of these results has to be questioned and evaluated.

### Impact on clinical application

As previously shown by different authors, sophisticated HRFT models seem to deliver more precise FT results when adjacent to eloquent gliomas. Also, our study group previously investigated not only the quantitative differences [4] between DTI- and QBI-results but also the quality of the processed tracts. We found, that QBI-FT provides lower offset values compared to intraoperative IOM results, suggesting more valid results [4].

The findings of the present study again support, that although QBI-FT requires longer processing times than DTI for both examined fiber tracts, with OR needing the longest (35 min vs. 22 min), it is still applicable in the clinical setting. Furthermore, the lower interrater variability, suggesting greater robustness indicates, that QBI-FT could be applied as neurosurgical standard in the future.

### Limitations

Previous studies already suggest that the utilization of HARDI techniques ensures a better reconstruction of white matter tracts in complex fiber crossing regions compared with DTI-FT. However, there is still a void in the literature examining the feasibility and reliability of FT and particularly HRFT techniques in the presence of intracerebral lesions. The main strength of the current study is that we are the first to examine the reproducibility and intra- and interrater variability for two important fiber bundles in the proximity of intracerebral lesions using conventional DTI-FT compared with a sophisticated model for HRFT under the special considerations of clinical data and applicability. However, some limitations do exist. First, we examined a relatively small cohort of patients and two selected fiber pathways. To corroborate the results, more raters with different levels of experience are necessary. A higher number of iterations for the FT procedure, also for other fiber pathways is mandatory. Other models for HRFT should be taken into account. The lesions’ histopathology was heterogeneous; however, to evaluate this influence on FT was beyond the scope of the study. The robustness of our results may be questionable because the study compared the fiber tracking of two raters with different levels of experience. However, our findings confirm that, independent of the experience level, the intra-rater agreement for DCS and JC was almost perfect for QBI-based FT and substantial for DTI-based FT for the reproducibility of the CST, while the inter-rater agreement was substantial for the QBI-based FT for both fibers and moderate for the DTI-based FT for both fibers. One might argue that the moderate inter-rater variability might be attributable to the different levels of experience. Nevertheless, at our institution, residents are trained from the first day of their residency in fiber tracking and anatomical landmarks. Since no robust evidence exists so far, we believe that our study makes a substantial contribution to the merit of preoperative planning, especially for lesions adjacent to critical white matter tracks, and its merit for young neurosurgeons. Although our findings suggest a higher robustness against user-dependence for QBI-FT, the given results do not answer the question on validity of the FT results.

## Conclusions

Our interrater-findings suggest that QBI-FT might be a more robust technique than DTI-FT with respect to user-dependence and disturbed areas of diffusion, while applicable for the neurosurgical clinical setting in terms of processing- and post-processing time and effort. Together with the findings on intrarater agreement regarding the reproducibility of both techniques, our results emphasize the impact of ROI placement when performing manual ROI segmentation or additional implementation of technical aids to detect eloquent cortical structures, at least when using DTI-FT. Further studies with a greater sample size and more raters are warranted to shed light on this topic.

## Data Availability

The datasets generated during and/or analyzed during the current study are available from the corresponding author on reasonable request.

## Declarations

### Authors and affiliations

Pavlina Lenga, Department of Neurosurgery, Heidelberg University Hospital, Heidelberg, Germany; Moritz Scherer, Department of Neurosurgery, Heidelberg University Hospital, Heidelberg, Germany; Peter Neher, German Cancer Research Center, Division of Medical Image Computing, Heidelberg, Germany; Jessica Jesser, Department of Neuroradiology, Heidelberg University Hospital, Germany; Irada Pflüger, Department of Neuroradiology, Heidelberg University Hospital, Germany; Klaus Maier-Hein, Department of Radiation Oncology, Heidelberg University Hospital, Heidelberg, Germany and German Cancer Research Center, Division of Medical Image Computing, Heidelberg, Germany; Andreas W. Unterberg, Department of Neurosurgery, Heidelberg University Hospital, Heidelberg, Germany; Daniela Becker, Department of Neurosurgery, Heidelberg University Hospital, Heidelberg, Germany.
